# Patient and clinician experiences and opinions of the use of a novel home use medical device in the treatment of peripheral vascular disease - a qualitative study

**DOI:** 10.1186/s13047-021-00496-2

**Published:** 2021-12-03

**Authors:** Charlotte E. Sedgwick, Charlotte Growcott, Shehnaz Akhtar, Daniel Parker, Erik Mulder Pettersen, Farina Hashmi, Anita Ellen Williams

**Affiliations:** 1 University of Salford, School of Health & Society, Brian Blatchford Building, Frederick Road Campus, Salford, M6 6PU UK; 2 Department of Surgery, Sørlandet Hospital, Kristiansand, Norway; 3 Department of Circulation and Medical Imaging, Faculty of Medicine and Health Sciences, Norwegian University of Science and Technology, Trondheim, Norway

## Abstract

**Background:**

Peripheral vascular diseases have a significant impact on functional quality of life. Previous research has demonstrated the complex, limiting and costly economic implications of these conditions such as lower limb ulceration chronicity and ischaemic amputation. These complex, limb and life threatening conditions demand the development of novel interventions with objective research as part of that development. Hence, a novel intermittent negative pressure medical device in the form of a wearable boot (FlowOx™) was developed. As part of the development process, this study aimed to explore patient and clinician opinions of the boot.

**Methods:**

A qualitative approach was used to collect patient and clinician experiences in Norway. An advisory group informed the semi-structured questions used in seven patient interviews and one clinician focus group (*n* = 5). The data were recorded digitally and transcribed verbatim. Patient and clinician data were analysed as distinct groups using a thematic process.

**Results:**

Data analysis resulted in five themes from the patients which gave insight into; the impact of the disease process; practicalities of using the boot, positive experiences of use; perceived outcomes; reflecting on use. Six themes were created from the clinicians. These gave insight into; ideal outcomes and how to measure them; ways to potentially use the boot; using research in healthcare; positives of the device; observed effects and next steps; potential improvements to the device.

**Conclusion:**

This study provides insight into the experiences and opinions of FlowOx™. Patients and clinicians were positive about the device due to its ease of use. Those patients with peripheral arterial disease experienced significantly more benefit, especially for ischaemic ulceration than those with a chronic venous condition. Clinicians placed value on the patient reported outcomes in the treatment decision-making process. This preliminary study into experiences of FlowOx™ use provides valuable feedback that will inform design modification and ongoing research into implementation points and prospective user groups. FlowOx™ demonstrates potential as a conservative therapy offering users a convenient, home use, self-care management solution for improving symptomatic peripheral arterial disease and quality of life.

**Supplementary Information:**

The online version contains supplementary material available at 10.1186/s13047-021-00496-2.

## Background

Peripheral arterial disease and chronic venous aetiological conditions are a global problem in an ageing population [1, 2] with peripheral arterial disease carrying high associated risks of cardiovascular morbidity and mortality [3–6]. Walking-associated claudication pain proves a frequent symptom of peripheral arterial disease, yet many are asymptomatic and consequently unaware of disease presence [7, 8]. Lower limb ulceration, gangrene or prolonged rest pain classifies as chronic limb-threatening ischaemia [9], which requires prompt revascularisation to prevent limb loss [9, 10]. Although widespread in its epidemiology, peripheral arterial disease remains largely underdiagnosed and hence undertreated [11–13]. The cost of treatments for ulceration alone continues to prove a significant economic burden and at a cost to developing countries that is estimated between 1 and 3% of their total health expenditure [14–16]. For the patient, lower limb vascular compromise has been shown to significantly affect quality of life by reducing everyday functional capacity and instilling a growing uncertainty regarding illness, treatment and long-term health status [17–21].

The number of patients who have chronic venous disease are predicted to increase significantly in the coming years and decades. The global burden of chronic venous disease uses approximately 2% of healthcare budgets, [22–24]. The disease progression rates are reported to be 4% [25, 26] and varicose vein procedures, are projected to increase by 60% by 2021 [27]. The venous leg ulcer is a common type of ulcer in the lower extremity [28]. Venous leg ulcers accounts for 70–80% of ulcers presenting for evaluation and treatment to a range of healthcare disciplines. The prevalence of venous leg ulcers is up to 2% of the population and, importantly, increases to 5% of individuals over the age of 65 years old [29, 30]. Europe has up to 2.2 million people affected by venus leg ulcers, and over 6 million individuals are affected in the United States [31]. Although appropriate wound care and compression therapy can be effective in healing, the recurrence rates are high, these can be as high as 50–70% at 6 months [30].

With a last reported chronic limb threatening ischaemia regional prevalence in Norway of 0.24 to 0.26% in 40–69 year olds [32], increased efforts have been placed on implementing health promotion strategies within identified at risk populations [33, 34]. However, there are no studies or reports that have described a recent change in the prevalence of chronic limb threatening ischaemia. In the Norwegian registry for vascular surgery (NORKAR), over the last 5 years an increased share of patients treated for chronic limb threatening ischaemia have peripheral arterial disease. It would be interesting to examine the context and evaluate the health strategies in depth.

Over the last two decades, Scandinavian studies show reductions in major amputation rates and increases in endovascular procedures, although Norwegian regional disparities occur [35–37]. In line with international trends [38], the studies reveal an improvement in diabetic foot health [37], increased chronic limb threatening ischaemia awareness, optimal treatment strategies with better organised and widespread vascular surgical services.

However, sub-optimal results for limiting deterioration have been found in relation to increasing risk-factor awareness and lifestyle modification [39–44]. Therefore, effective and acceptable therapies are needed to improve circulation and promote wound healing. Self-management through targeted behavioural change proves a central element in vascular risk management [45]. Exercise programmes yield improvements in pain-free walking which may improve quality of life [46], still these prove unsuitable with active ulceration and are hindered by poor uptake and accessibility [47].

**Fig. 1:**
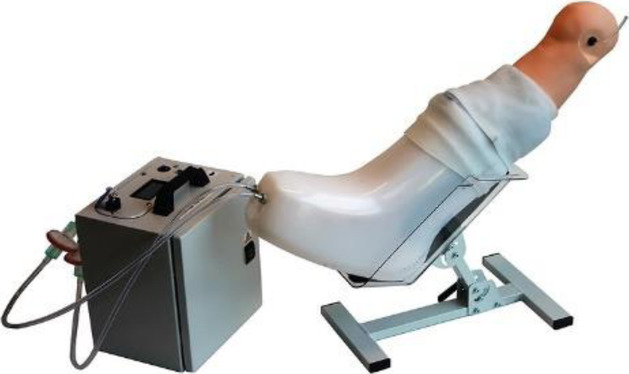
FlowOx™ device and components (image courtesy of Otivio AS, Oslo, Norway)

The use of directly applied negative pressure wound therapy has shown some improvements for chronic wounds [48, 49]. Negative pressure wound therapy has successfully been applied in a primary and home-care setting [50]. The introduction of a Norwegian hospital wound support team network has improved the clinical efficacy of the home care services and reduced the need for consultations at the hospital [51]. This model of home intervention, supported by a hospital network team, can be applied to other devices [50]. The recent development of a novel device (FlowOx™ *Otivio, Norway*, Fig. 1), that applies intermittent negative pressure to the lower leg but not directly to the wound, has demonstrated improvements in both micro and macro-circulation and improved healing in hard-to-heal wounds [51, 52]. The positive effects of intermittent negative pressure have been described to be caused by an increased arteriovenous pressure gradient, shear rate, and blood flow fluctuations, leading to improved endothelial function [53, 54]. Data published after this qualitative study was undertaken, has revealed improvements in pain-free walking distance after 12 weeks of intermittent negative pressure-treatment [55] and in a follow-up study of 24 weeks of intermittent negative pressure-treatment, both pain-free and maximum walking distance increased [56]. FlowOx™ is a CE marked medical device intended for use by patients with peripheral vascular disease. Such medical devices are becoming more known for home use; therefore safety, suitability and acceptability must be established [57, 58]. Therefore, this qualitative study aimed to gather patient experiences of using this self-use intermittent negative pressure medical device in relation to its usability, its design and value. Further, it aimed to gather clinician opinion on the clinical effects and benefits, financial benefits and critical price points in Norway healthcare.

## Methods

### Ethics

This qualitative study was granted ethical approval by the University of Salford, School of Health Sciences Ethics Committee (HSR1617–31) and the Regional Committees for Medical and Health Research Ethics, Norway.

### The intervention

FlowOx™ (Fig. 1) provides an intermittent negative pressure to the lower limb and foot in the form of a wearable boot. The leg and foot are suspended inside and held with medical grade pads. A silicone seal is pulled over the leg to allow a negative pressure to be established. The pressure is set and cycled intermittently with input from the control box. The system was prescribed by clinicians for use by patients for a recommended 2 hours use per day, however, this was not controlled, and some participants used the device for longer than 2 hours. At the point of data collection the system was provided for patients to use through funded research with some treatment costs paid by the company and grant funding (Research Reimbursements). Continued use of FlowOx™ beyond the research was paid for by patients, in some cases this cost was supplemented by the Norwegian healthcare system which provides an individual allowance for medical expenses.

### Participants and recruitment

The FlowOx™ treatment was not widely prescribed, therefore a purposive sample of patients and clinicians with experience of its use across two hospital sites in Norway (Oslo & Trondheim) were invited to participate. Potential participants who had used the device for a minimum of 6 months were given information sheets detailing the aim and the voluntary nature of the study. Informed consent was taken from patients prior to interview and from clinicians prior to the focus group.

Seven patients agreed to participate in an interview (Table 1). Two patients were solely Norwegian speaking, whilst five agreed to an English-speaking interview. An interpreter was present during interviews to eliminate unforeseen language barriers. Five clinicians from vascular clinical teams at two hospital sites were invited to participate in a focus group. Clinicians were members of vascular surgical staff (*n* = 3) and clinical research (*n* = 1) from Oslo and vascular surgical staff (*n* = 1) from Trondheim. Anonymity was achieved by using participant numbers during data collection and analysis. All audio files, transcripts, data analysis and data containing patient and clinician information were stored on a password protected server at the University of Salford.

**Table 1:** Patient demographics

**Patient**	**1**	**2**	**3**	**4**	**5**	**6**	**7**
**Gender**	Female	Male	Male	Female	Female	Male	Male
**Vascular Medical Notes**	Previous arterial ulcerations Peripheral arterial disease	Intermittent Claudication Peripheral arterial disease	Intermittent Claudication Peripheral arterial disease	Current venous ulceration	Previous arterial ulceration. Amputee. Peripheral arterial disease	Intermittent Claudication. Previous arterial ulceration. Peripheral arterial disease	Current venous ulceration.
**Place of intervew**	Own home Oslo	Own home Oslo	Meeting room Oslo	Meeting room Trondheim	Meeting room Trondheim	Meeting room Trondheim	Meeting room Trondheim

### Data collection

An advisory group for this qualitative study was formed and comprised of members of the main study which focussed on quantitative outcomes of the intervention [59]. Members included the Principal Investigator (FH), the quantitative research team (DP, SA) and qualitative researchers (AW and CS) informed the semi-structured questions for the face to face patient interviews aimed at understanding patient experiences and opinions on the usability of FlowOx™. The group also informed the focus group questions that sought to gain clinician opinions about the effectiveness of FlowOx™ as a treatment for peripheral arterial disease and/or ulceration, including cost implications.

Patient interviews were conducted at the participants convenience either at the hospital sites or in their own homes. Interviews were carried out by the main researcher (CS), a UK Podiatrist and a research assistant with clinical experience of patient interviews and management of lower limb vascular conditions. Question topics explored patients experiences of use, benefits and barriers, thoughts on design and cost value, additional treatments and recommendations for other users.

The clinician focus group was conducted at the Oslo hospital site and facilitated by the main researcher (CS). The choice of a focus group at the Oslo hospital was pragmatic to allow staff to contribute whilst being available for emergencies. Question topics explored experiences of clinical effects and benefits, research reimbursements and critical price points in Norway healthcare.

Data were collected with digital audio recording (*WS-853 Digital Voice Recorder, Olympus*) and supplemented with field notes. Patient interviews lasted between 12 and 34 min and the clinician focus group lasted 42 mins. The recorded data were translated from Norwegian to English as applicable and then transcribed verbatim using an independent service (*Go Transcript, UK*).

### Data analysis

**Fig. 2:**
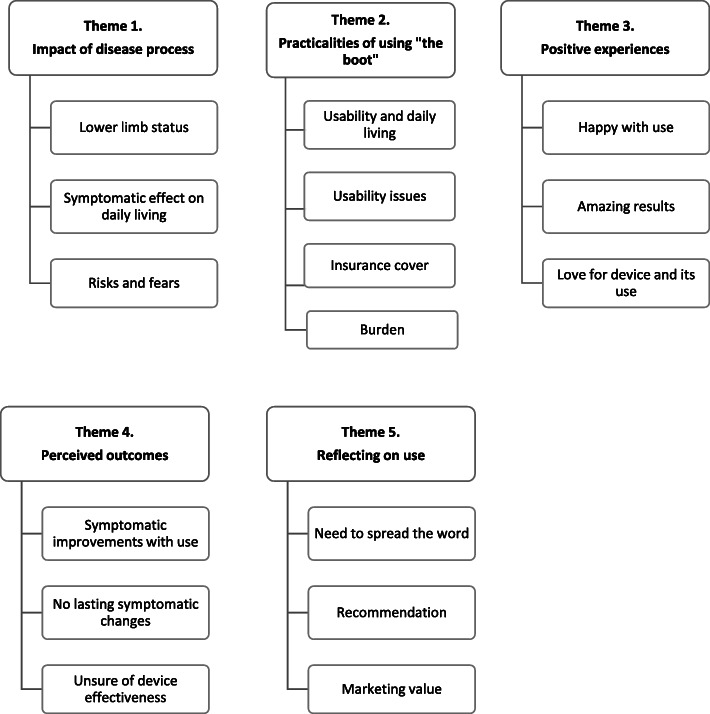
Patient Interviews: Primary and secondary themes

**Fig. 3:**
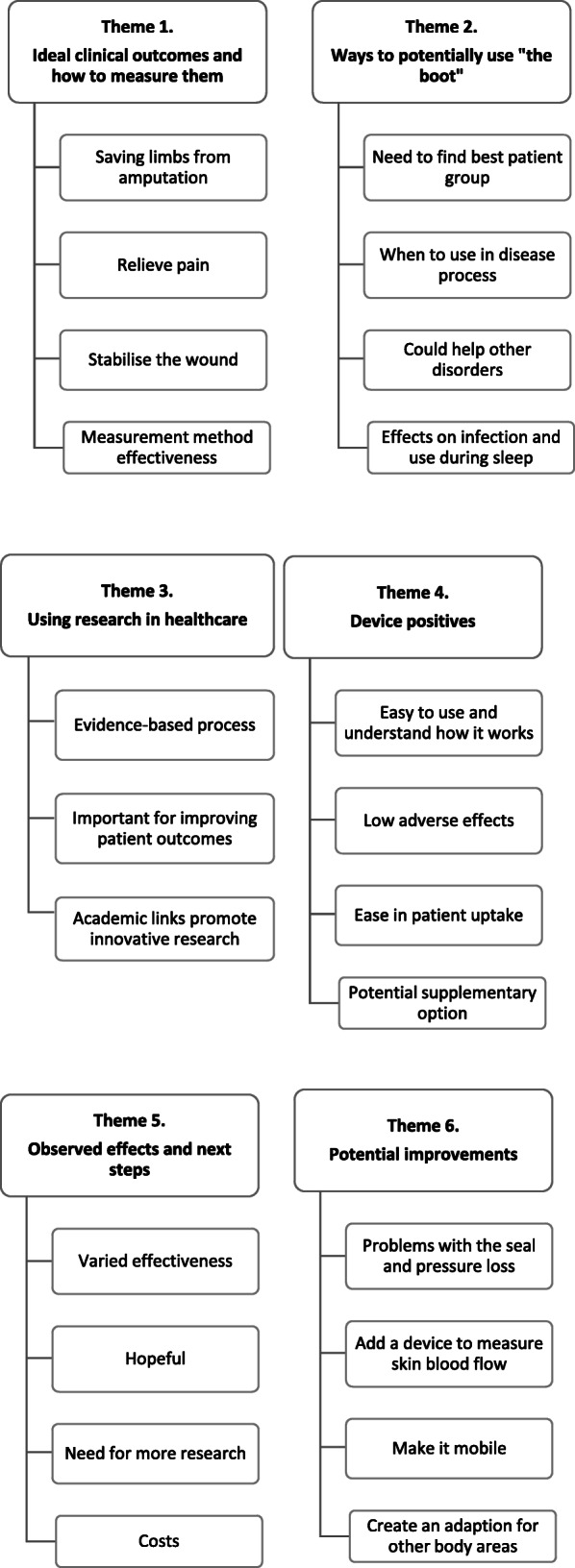
Clinician Focus Group - Primary and secondary themes

A pragmatic qualitative descriptive approach [60] with thematic analysis [61] was utilised. The analytical researchers (CS and CG) familiarised themselves with the data and generated initial codes by listening to audio recordings and re-reading interview and focus group transcripts. The addition of a second researcher (CG) to the initial analysis added to the credibility of the findings. Nvivo (*Nvivo 12 Pro, QSR International*) provided a processing platform for analysis. Emerging themes were then developed and refined through an individual review process that sought to underpin central concepts, reflect the descriptive and explicit content of the data [61] and validate results from participants transcripts. A naturalistic approach allowed researchers to gain rich descriptive experiences and opinions of the medical device used in the natural environment herein, the “home” [60, 62]. To reduce subjectivity and enhance rigour, the main researcher (CS) also undertook the analysis of focus group data with the second researcher (CG). An experienced qualitative senior researcher authors (AW) verified the analysis and results and then consensus was achieved. Extracts from the transcripts were used to illuminate the themes and to demonstrate truthfulness Figs. 2 and 3.

## Results

### Patient interviews

#### Theme 1. Impact of the disease process

Experiences of using FlowOx™ revealed recurrent dialogue from patients that focused on symptomatic changes to lower limb health. Personal accounts highlighted significant functional daily living changes from diagnosis and through disease progression. Pain levels dictated physical activity limits:

*“I could walk for some distance, and then I had to stop because it hurt so much.” [p3]*

The experience of pain also affected the quality of sleep:

*“It was like I put my toes in hot water. So, I had to go get up once every hour each night to walk around, slow it down … ” [p6]*

Most described treatment for ulceration and were within various stages ranging from static healing to complete resolution.

For the two participants with current venous ulceration they reported discomfort as p4 reveals,

*“ … I get this nagging feeling in my leg which is uncomfortable and painful when the dressing sticks to it.”*

Dialogue revealed how vascular compromise with peripheral arterial disease led to clinician discussion of the risks of amputation:

*“There is the danger that I may lose a leg … ” [p1]*

The risk of limb loss for those with peripheral arterial disease ultimately led to experiencing feelings of fear and worry:

*“So, I was afraid that I was going to lose both of them.” [p5]*

*“To go from being physically fit to talking about an amputation in a course of six months did things with me. It did do things with me mentally.” [p6]*

#### Theme 2. Practicalities of using the “boot”

Description and demonstrations of how practical the device was to use emerged as a prominent theme. Most found the device comfortable and easy to use:

*“Nothing difficult, ‘cause everything was easy.” [p4]*

All were able to use the device independently:

*“It’s an easy device to use. Just put it on and press the button and forget about it.” [p2]*

Some practical usage issues were revealed with the seal component. Pulling on the seal proved difficult for some. It was postulated that the elderly may have difficulties with the design.:

*“So there is some difficulty getting hold of it to get it over there...” [p3]*

*“I think that elderly people will not manage to use this without having home care or something similar … ” [p7]*

Differences in the seal material lead to an increase in size, corrosion and problems achieving maximum pressure:

*“It started failing because it just got a little bigger and bigger.” [p1]*

*“The machine shows leakage.” [p2]*

Views on whether personal health insurance would cover costs for this novel device were mixed and with one who was unsure:

*“I don't know. I haven't asked. But I really hope they will do it, because I think many people can have another life with it.” [p2]*

During device use, patients’ experienced various sensations within the leg and foot described as tingling and pain relief:

*“And if I did feel discomfort, a lot of discomfort, it was the device that I actually used. So, if I really was having a lot pain, I used to put my foot into the boot and it did have an effect. Because it wasn't painful … When it was bad it was what I used to ease and comfort.” [p5]*

The time taken to use the device, its inability to be a mobile device, the space taken to accommodate it and its sound became a burden for some:

*“Three hours a day is three hours a day. And it's three hours missed to do something else.” [p4]*

All described accommodating the device in their homes and around routines. One patient described using the device for longer than the prescribed daily 2 hours:

*“ … I was a little bit desperate at that time, so I was willing to push the boundaries probably. Okay, you know and I did use, I have probably overused it … I took control of it. I did. And being able to decide for myself that I was going to overuse the boot really was a decision that I'm quite happy that I did as well. Without actually knowing the technical or the medical information behind it. It was a chance I took.” [p6]*

#### Theme 3. Positive experiences

Positive experiences from device use emerged throughout most interviews:

*“I am a 100% happy up to now … I have nothing negative.” [p6]*

Two patients gave lengthy detail to the changes to their lower limb health during device use and that had resulted in a complete symptomatic reversal, varying degrees of wound healing and complete wound resolution:

*“ … I understand that it was this block in the vein that was coming out. That was amazing … my usual doctor she couldn’t understand what’s happening. She was just wow … And the feeling was fantastic, too, because then I realised that my toes was safe.” [p6]*

*“I'm warm and good in my leg and my foot which was dead.” [p1]*

Some described fond feelings for the device and relief in avoiding amputation. Continued use was described as necessary to prevent symptom deterioration:

*“So now it's not repairing but keeping it as it shall be.” [p4]*

*“A little week without and my foot is going to be bigger.” [p7]*

These positive health improvements were described as having a significant impact on the ability to carry out daily tasks:

*“I couldn't walk … I had to use the wheelchair all the time, but now I can walk, I can do different things. “I am more like a normal person." [p2]*

*“I do everything which I could do before, so, my life quality has been very improved through this project.” [p5]*

#### Theme 4. Perceived outcomes

Outcomes included improved healing, circulation, and pain reduction.

*“I think the healing may have come quicker because of it. I cannot say why, but, why I think so, but that's what I feel. I feel that everything … all the wounds were healed, eventually. And I think that healing process was a little bit quicker because of that.” [p6]*

However, dialogue emerged from one patient with chronic venous ulceration experiencing minimal improvement:

*“Maybe in the start in the first 14 days, my head thinks … It was a little tiny better, but then it all stopped.” [p4]*

One patient remained unsure as to whether observed improvements were due to device use alone:

*“I wish I had the information to say it was the boot that did that. I don't have that, and I don't know if anybody does, to be honest. If you take it in terms of where I was … and what was then introduced in my treatment from that period and after. There was only one change in my treatment, and that was the usage of the boot. It wouldn't be wrong to insinuate that the boot has had a very positive effect, if all, to my situation.” [p6]*

For those with some or minimally perceived benefit, there remained a level of optimism for further use:

*“I want it to work.” [p5].*

#### Theme 5. Reflecting on use

The participants recommendations were a reflection on the potential value of device for others:

***“****Well, as time goes by one is willing to try almost anything. I could not guarantee they would benefit from it, but I would be recommending it because of what experience that I've had.” [p1]*

With current recommendation being implemented through social media, chat forums and casual discussion:

*“There is a lot of people out there that have big problems. Big, big problems. I have spoken to them, and I have posted up my treatment. The thing that has always pushed forward which is the difference, which they don't have out there, is this treatment boot. There is interest for it. And people will, when it comes to your life, it is about being functional. If you're not functional it just ‘disabilitates’ everything else that's in your life.”[p4]*

Views on how much they were willing to pay along with marketing suggestions for the device were mixed:

*“So monetary wise it’s certainly is worth a lot in terms of improving people’s functionality in their everyday life … it is a foot saver, you could say.” [p5]*

*"I call it the wonder machine."[p4]*

*“I can't set a price on it because it saved my life. It gave me my life back, in a way, so for me it's … priceless … one suck and you're hooked.” [p1]*

### Clinician focus group

#### Theme 1. Ideal clinical outcomes and how to measure them

Clinician experiences from prescribed use revealed a range of desired clinical outcomes, specifically saving limbs, relieving pain and wound stabilisation:

*“I think it’s limb salvage … to save limbs.” [p5]*

*“I think the pain scale of the patient is an important aspect of this, if this improves the pain from the ulcer in any way, simply for the QoL for the patient.” [p2]*

*“ … stop the progression of the ischaemic wound.” [p1]*

Clinicians described clinical methods used for measuring device efficacy and effectiveness from wound size, pain scales and blood flow. Some gave detailed accounts as to how traditional clinical methods used to measure blood flow may not provide efficacious results:

*“There is a study where they have done training on claudicants, where they have done an exercise program and where they have clinically a longer walking distance, which with no benefit at the ankle-brachial index for example. So, it’s an indication that maybe these measurements are not done necessarily reflect the clinical reality.” [p2]*

*“If you have wound healing and all the other parameters will tell you that there is no change, I would still continue using the boot.” [p4]*

Clinicians suggested that assessing small vessel blood flow may be more indicative of device effects:

*“If you’re looking for a minimal effect it could maybe be to measure skin blood flow with laser doppler.” [p3]*

*“The boot has not necessarily an effect on the big vessels, it's more on micro level.” [p5]*

#### Theme 2. Ways to potentially use “the boot”

Opinions revealed a need to find the best patient group for the device:

*“I think hopefully over time we'll be able to select the patient group that will have a benefit from this rather than all of them, because I don't think everybody will have a benefit of the boot.” [p2]*

The potential need to discover when in the disease process to use the device was also suggested. One comment highlighted that perhaps the trials happening now were too late:

*“Also, since this is a new equipment, there is a chance that some of the patients have come too far in the process. It's important to be aware that they’re maybe too late. In my opinion, many of the patients we have tested it on so far, it's actually too late, so then it would end in amputation anyway.” [p3]*

In contrast, the device was also implicated as having the potential to provide a last option for patients:

*“I think that the FlowOx™ boots could be a promising supplement to patients who basically have reached the end of the line.” [p2]*

Clinicians postulated that the device has potential to be used with other patient groups, such as those with vasospastic disorders and pressure ulceration. It was also suggested that device effect on infection and safe use during sleep could be further investigated:

*“The treatment of antibiotics could also in theory have been more effective using this system, if you, in that way, can get the antibiotics closer to the bacteria in the wound by using the system … ” [p3]*

#### Theme 3. Using research in healthcare

Research in Norwegian healthcare was described as requiring rigorous testing of new medical interventions to provide the evidence to secure funding:

*“What we need to work on is developing new methods maybe in a high-volume centre and then when this is proven, try and get it out to the more peripheral areas.” [p2]*

Research was highlighted as essential to improve outcomes for patients:

*“We have to try out new equipment and new devices and be in the research to see if we can improve the outcome for our patients where there is no other hope for them.” [p1]*

The practicalities of facilitating new research in hospitals and with novel interventions was reported by clinicians as more receptive in those hospitals with academic links:

*“It depends on the hospital region because some are research facilitated or has connection to the University, such as this hospital, who are more inclined to break barriers, try out new things, do more research.” [p3]*

#### Theme 4. Device positives

Positive opinions of device implementation and uptake revealed its ease of use, simple instructions and low adverse effects:

*“I just want to add that it's fairly easy for patients to understand how this works in theory and it's easy for us, as clinicians, to convince patients to try it because of the adverse effects are very small. It's easy for them to administer at home. It's easy for them to be positive in terms of wanting to try this. I think that's a huge positive in terms of getting more experience in using the boot.” [p5]*

The device was suggested as a good potential supplementary option for patients unable to engage in alternative therapies:

*“We know that supervised exercise training is the best, but the problem is that it's not easy to get the patients do it. It's not always even our option around, so I think this maybe something that could be added early on. If that's possible, that would be great.” [p2]*

*“I like the theoretical background on this device and also seen some good clinical results. As a summary, I'm going to say that it could be a good supplementary device for treating patients with ischemia.” [p1]*

#### Theme 5. Observed effects and next steps

Clinicians described seeing varied effectiveness from patient use, yet remained hopeful:

*“We have had two patients who had very good effect of the device. Then we have two patients with intermittent effect of the device and two patients without any effect of the device.” [p4]*

It was stated throughout the focus group that there was a need for more research as numbers were limited:

*“We really need more data to say if there is a proved effect or not.” [p2]*

When discussing price points, a high price was said to be unrealistic. There were suggestions that Government subsidies should shoulder medical device costs along with low contributions from patients, as this was considered an affordable solution that could benefit more people:

*“Our patients, they don't have that much money, so the price could not be very high.” [p3]*

*“I think as well, you need to put this in health benefits frame and maybe it would be better to have a lower price so that it could be used over a larger area rather than it be limited by a high price range.” [p4]*

The high cost of amputation was highlighted and how limb salvage should be a priority when considering research into new medical devices:

*“Now, we're back to the limb salvage thing because that's very important because if you are saving a limb, it's much less cost than if you have to amputate.” [p5]*

#### Theme 6. Potential improvements

The final theme reveals a small number though important opinions on design improvements. Problems arose with the seal which resulted in a loss of controlled pressure:

*“The problem has been this silicone, as you call it, that has been porous and patients get leak in use … ” [p4]*

There was consensus that it would be beneficial if the device could be designed for the patient to be able to move around with the device on rather than having to sit, as p3 explains:

*“You have to sit still in a way for a long time. If you can move around, it would be more easy to use it.” [p3]*

Other suggested improvements included adding a device to measure skin blood and creating an adaption for use on other body parts.

## Discussion

This study has provided insight into patient and clinician experiences and opinions on the use of this novel intermittent negative pressure device that will add descriptive perspectives to the current evidence base and product development. The results also demonstrate the complex interplay and overlap between the themes particularly in relation to the patient’s positive experiences and perceived outcomes.

Detailed accounts from patients of changes to their health and lifestyle over disease duration were forefront throughout interviews. The experience of pain, as well-documented in lower limb vascular compromise, was the focal debilitating factor in patients’ sleep quality and mobility [20, 63–65]. From these personal accounts of lower limb deterioration, there was clear unease and fear of amputation; highlighting the significant implications of physical health impacting mental wellbeing [66, 67]. These life events led patients to take an active incentive in preventing further personal deterioration and which facilitated ease in intervention uptake through shared decision-making.

The clinicians agreed that amputation was the last resort and that limb salvage was the ultimate primary outcome for any intervention such as the FlowOx™. When gathering evidence for such outcomes, the use of ankle-brachial pressure index as a clinical marker of vascular status was posited as failing to give results that were consistent with patient reported improvements. This aligns with a systematic review of exercise programmes for intermittent claudication which increase pain-free walking distances, yet without ankle-brachial pressure index improvements [46]. Here, intervention efficacy is evidenced through subjective patient reported outcomes. Patient reported outcomes are increasingly implemented as a measure of intervention effectiveness and value in treatment decision-making [68, 69]. When considering their own patient reported outcomes, clinicians’ placed value on these as a valid rationale for continuing device use. However, the need for objective outcome measures alongside patient reported outcomes, would provide clear evidence for the efficacy of this treatment. Data concerning the clinical efficacy of the FlowOx™ is currently being compiled for publication. This will allow for comparisons to be made between patient reported outcomes and objective measures.

Domestic layout changes and problem solving with device parts for successful usability displayed active patient participation and engagement. Experiences of malfunction with the seal were a source of frustration, however, device use positives that conveyed ease of use, low adverse effects and perceived symptomatic resolution facilitated acceptability for both patients and clinicians. Patients who were more physically able reported a burden of “lost time” during use, which was associated with the inability to mobilise. Here exercise programmes may be a more suitable treatment for these patients, along with FlowOx™ as an adjunct. In contrast, FlowOx™ proves advantageous in providing a replacement therapy for those less able to ambulate or attend walking programmes. When considering such benefits and burden, limitations exist for direct negative pressure wound therapy and its effects on quality of life during the initial use phase, including malodour, increased pain and anxiety; yet QoL is said to improve at therapy end [70]. Early adverse effects of direct negative pressure wound therapy and intermittent negative pressure use have the potential to affect compliance, thus determining whether the prescribed therapeutic period is completed for benefit gain. The participants experiencing INP delivered through FlowOx™ saw benefits with time and without any of the limitations reported in initial use negative pressure wound therapy. As physical activity and exercise programmes remain the main therapy for peripheral arterial disease and as a modifiable risk for coronary vascular disease [71]; both patients and clinicians suggested design modifications that would allow for some degree of movement and increased usability.

Patient opinions on perceived effects from use were mixed, with two experiencing complete symptomatic resolution, two resolved but unsure if it was the device, one improved and two minimal/no improvement. These varied effects from device use were reciprocated with clinician reports. Patients who had chronic venous conditions experienced less benefit from use when compared to those with peripheral arterial disease.

Clinicians had mixed opinions as to what point in the disease process would be optimal to implement the device. These opinions ranged from use in early diagnosis, as an adjunct or last resort. There was however a consensus for the need to find the most appropriate patient group through further research involving more participants. An existing evidence base for FlowOx™ [51–53, 55, 56], along with observed benefits from patients were well regarded. As researchers, clinicians and medical educationalists, they felt it ethically appropriate to implement novel and evidence-based interventions into their clinics. This was considered vital for those patients having exhausted invasive treatment options that would inevitably place the emphasis back on conservative management [72].

Both patients and clinicians remained hopeful for others to gain benefit from use, whether facilitated through continued research, health insurance or with Government funding. Those patients reporting perceived benefit actively sought to recommend the device and give hope to others by reaching through social media, support groups and in casual conversation. The value for those having perceived benefits of limb salvage and as clinicians described, the subsequent societal costs savings, along with patient reported outcomes in shared decision making, highlight the vital importance of patient voices in patient centred care [73]; though the positive benefits identified in this study require further research. Recent published cost-effective analysis, comparing FlowOx™ home treatment to standard hospital care, has demonstrated a cost advantage in favour of the home treatment depending on the severity of the disease [74].

Researchers recognise a lack of common language between patients and interviewer may be a limitation to the study. This was addressed through the presence of an interpreter checking meaning with each patient and outsourced translation transcribing. We are mindful of the small number of participants, mixed vascular conditions and limited demographics. However, this study was exploratory and aimed to gain insight, understanding and meaning from these purposively sampled participants rather than to achieve generalisability. To add to this Norwegian perspective, it is recommended that patient and clinician experiences and opinions of FlowOx™ are ascertained in UK health services.

## Conclusions

Peripheral vascular diseases have a significant impact on a person’s functional quality of life. The complex, limiting and costly implications of these conditions now makes novel intervention research essential. Whilst using FlowOx™, patients with peripheral arterial disease experienced significantly more benefit, especially for ischaemic ulceration than those with a chronic venous condition. Clinicians placed value on patient reported outcomes in the treatment decision-making process. This preliminary study into experiences of FlowOx™ use provides valuable feedback that will inform design modification and ongoing research into implementation points and prospective user groups.

Exercise, optimal medical treatment and adequate revascularisation strategies are the recommended state-of-the-art treatment for chronic limb threatening ischaemia. However, some patients continue to have arterial insufficiency after maximal efforts to revascularise the limb. These patients often have wounds and comorbidities which are an obstacle to exercise treatment. FlowOx™ may be a suitable option for such patients as a conservative therapy that offers a convenient, home use, self-care management solution for improving symptomatic peripheral arterial disease and quality of life.

## Supplementary Information

**Additional file 1.** .

## Data Availability

Transcripts are available on request.

## References

[1] Dua A, Lee CJ (2016). Epidemiology of peripheral arterial disease and critical limb ischemia. Tech Vasc Interv Radiol.

[2] Xie T, Ye J, Rerkasem K. The venous ulcer continues to be a clinical challenge: an update. Burn Trauma. 2018;6. 10.1186/s41038-018-0119-y. 10.1186/s41038-018-0119-y PMC6003071 29942813

[3] Norgren L, Hiatt WR, Dormandy JA, Nehler MR, Harris KA, Fowkes FGR (2007). Inter-society consensus for the Management of Peripheral Arterial Disease (TASC II). J Vasc Surg.

[4] Hirsch AT, Duval S (2013). The global pandemic of peripheral artery disease. Lancet.

[5] Fowkes FGR, Aboyans V, Fowkes FJI, McDermott MM, Sampson UKA, Criqui MH (2017). Peripheral artery disease: epidemiology and global perspectives. Nat Rev Cardiol.

[6] Shu J, Santulli G (2018). Update on peripheral artery disease: epidemiology and evidence-based facts. Atherosclerosis.

[7] Signorelli SS, Vanella L, Abraham NG, Scuto S, Marino E, Rocic P (2020). Pathophysiology of chronic peripheral ischemia: new perspectives. Ther Adv Chronic Dis.

[8] Jensen SA, Vatten LJ, Romundstad PR, Myhre HO (2003). The prevalence of intermittent claudication. Sex-related differences have been eliminated. Eur J Vasc Endovasc Surg.

[9] Conte MS, Bradbury AW, Kolh P, White JV, Dick F, Fitridge R (2019). Global vascular guidelines on the management of chronic limb-threatening ischemia. J Vasc Surg.

[10] Morley RL, Sharma A, Horsch AD, Hinchliffe RJ (2018). Peripheral artery disease. BMJ.

[11] Andras A, Ferket B (2014). Screening for peripheral arterial disease. Cochrane Database Syst Rev.

[12] Tehan PE, Fox M, Stewart S, Matthews S, Chuter VH. Lower limb vascular assessment techniques of podiatrists in the United Kingdom: a national survey. J Foot Ankle Res 2019 121 2019;12:1–12. 10.1186/S13047-019-0341-2. 10.1186/s13047-019-0341-2 PMC6530165 31139263

[13] Jelani Q, Petrov M, Martinez SC, Holmvang L, Al-Shaibi K, Alasnag M (2018). Peripheral arterial disease in women: an overview of risk factor profile, clinical features, and outcomes. Curr Atheroscler Rep.

[14] Guest JF, Ayoub N, McIlwraith T, Uchegbu I, Gerrish A, Weidlich D, Vowden K, Vowden P (2017). Health economic burden that different wound types impose on the UK’s National Health Service. Int Wound J.

[15] Guest JF, Vowden K, Vowden P (2017). The health economic burden that acute and chronic wounds impose on an average clinical commissioning group/health board in the UK. J Wound Care.

[16] Olsson M, Järbrink K, Divakar U, Bajpai R, Upton Z, Schmidtchen A, Car J (2019). The humanistic and economic burden of chronic wounds: a systematic review. Wound Repair Regen.

[17] Maddox D (2012). Effects of venous leg ulceration on patients’ quality of life. Nurs Stand.

[18] Maksimovic M, Vlajinac H, Marinkovic J, Kocev N, Voskresenski T, Radak D (2014). Health-related quality of life among patients with peripheral arterial disease. Angiology.

[19] Gorely T, Crank H, Humphreys L, Nawaz S, Tew GA (2015). “Standing still in the street”: experiences, knowledge and beliefs of patients with intermittent claudication-a qualitative study. J Vasc Nurs.

[20] Galea Holmes MN, Weinman JA, Bearne LM (2017). You can’t walk with cramp!’ A qualitative exploration of individuals’ beliefs and experiences of walking as treatment for intermittent claudication. J Health Psychol.

[21] Abaraogu UO, Ezenwankwo EF, Dall PM, Seenan CA (2018). Living a burdensome and demanding life: a qualitative systematic review of the patients experiences of peripheral arterial disease. PLoS One.

[22] Evans CJ, Fowkes FG, Ruckley CV, Lee AJ (1999). Prevalence of varicose veins and chronic venous insufficiency in men and women in the general population: Edinburgh vein study. J Epidemiol Community Health.

[23] Rabe E, Guex JJ, Puskas A, Scuderi A, Fernandez QF (2012). Epidemiology of chronic venous disorders in geographically diverse populations: results from the vein consult program. Int Angiol.

[24] Rice JB, Desai U, Cummings AK, Birnbaum HG, Skornicki M, Parsons N (2014). Burden of venous leg ulcers in the United States. J Med Econ.

[25] Lee AJ, Robertson LA, Boghossian SM, Allan PL, Ruckley CV, Fowkes FG (2015). Progression of varicose veins and chronic venous insufficiency in the general population in the Edinburgh vein study. J Vasc Surg Venous Lymphat Disord.

[26] Pannier F, Rabe E (2012). The relevance of the natural history of varicose veins and refunded care. Phlebology.

[27] Onida S, Davies AH (2016). Predicted burden of venous disease. Phlebology.

[28] Eberhardt RT, Raffetto JD (2014). Chronic venous insufficiency. Circulation.

[29] Chi YW, Raffetto JD. Venous leg ulceration pathophysiology and evidence based treatment. Vasc Med (United Kingdom) 2015;20:168–181. 10.1177/1358863X14568677, 2. 10.1177/1358863X14568677 25832604

[30] O’Donnell TF, Passman MA, Marston WA, Ennis WJ, Dalsing M, Kistner RL (2014). Management of venous leg ulcers: clinical practice guidelines of the Society for Vascular Surgery® and the American venous forum. J Vasc Surg.

[31] Broszczak DA, Sydes ER, Wallace D, Parker TJ (2017). Molecular aspects of wound healing and the rise of venous leg ulceration: omics approaches to enhance knowledge and aid diagnostic discovery. Clin Biochem Rev.

[32] Jensen SA, Vatten LJ, Myhre HO (2006). The prevalence of chronic critical lower limb ischaemia in a population of 20,000 subjects 40-69 years of age. Eur J Vasc Endovasc Surg.

[33] O.E.C.D (2016). OECD Health Policy overview: Health Policy in Norway.

[34] O.E.C.D (2015). OECD Cardiovascular Disease and Diabetes: Policies for Better Health and Quality of Care.

[35] Wendt K, Kristiansen R, Krohg-Sørensen K, Gregersen FA, Fosse E (2017). Norwegian trends in numbers of lower extremity revascularisations and amputations including regional trends in endovascular treatments for peripheral arterial disease: a retrospective cross-sectional registry study from 2001 to 2014. BMJ Open.

[36] Witsø E, Lium A, Lydersen S (2010). Lower limb amputations in Trondheim, Norway: a 40% reduction in diabetic major lower-limb amputations from 1996 to 2006. Acta Orthop.

[37] Jørgensen ME, Almdal TP, Færch K (2014). Reduced incidence of lower-extremity amputations in a Danish diabetes population from 2000 to 2011. Diabet Med.

[38] Carinci F, Uccioli L, Massi Benedetti M, Klazinga NS (2020). An in-depth assessment of diabetes-related lower extremity amputation rates 2000–2013 delivered by twenty-one countries for the data collection 2015 of the Organization for Economic Cooperation and Development (OECD). Acta Diabetol.

[39] Willigendael EM, Teijink JAW, Bartelink ML, Boiten J, Moll FL, Büller HR, Prins MH (2004). Peripheral arterial disease: public and patient awareness in the Netherlands. Eur J Vasc Endovasc Surg.

[40] Coughlin P, Gulati V, Mavor A, Gough M, Homer-Vanniasinkam S (2007). Risk factor awareness in patients with peripheral arterial disease. J Cardiovasc Surg (Torino).

[41] Muthu C, Chu JJ, Le Heron C, Roake JA, Lewis DR (2007). Patient awareness of risk factors for peripheral vascular disease. Ann Vasc Surg.

[42] Cunningham MA, Swanson V, Pappas E, O’Carroll RE, Holdsworth RJ (2014). Illness beliefs and walking behavior after revascularization for intermittent claudication: a qualitative study. J Cardiopulm Rehabil Prev.

[43] Lokin JLC, Hengeveld PJ, Conijn AP, Nieuwkerk PT, Koelemay MJW (2015). Disease understanding in patients with intermittent claudication: a qualitative study. J Vasc Nurs.

[44] Wann-Hansson C, Wennick A (2016). How do patients with peripheral arterial disease communicate their knowledge about their illness and treatments? A qualitative descriptive study.(report). BMC Nurs.

[45] Lovell M, Myers K, Forbes TL, Dresser G, Weiss E (2011). Peripheral arterial disease: application of the chronic care model. J Vasc Nurs.

[46] Lane R, Harwood A, Watson L, Leng GC. Exercise for intermittent claudication. Cochrane Database Syst Rev. 2017;2017(12). 10.1002/14651858.CD000990.pub4. 10.1002/14651858.CD000990.pub4 PMC6486315 29278423

[47] Makris GC, Lattimer CR, Lavida A, Geroulakos G (2012). Availability of supervised exercise programs and the role of structured home-based exercise in peripheral arterial disease. Eur J Vasc Endovasc Surg.

[48] Vig S, Dowsett C, Berg L, Caravaggi C, Rome P, Birke-Sorensen H, Bruhin A, Chariker M, Depoorter M, Dunn R, Duteille F, Ferreira F, Martínez JM, Grudzien G, Hudson D, Ichioka S, Ingemansson R, Jeffery S, Krug E, Lee C, Malmsjo M, Runkel N, Martin R, Smith J, International Expert Panel on Negative Pressure Wound Therapy [NPWT-EP] (2011). Evidence-based recommendations for the use of negative pressure wound therapy in chronic wounds: steps towards an international consensus. J Tissue Viability.

[49] Yao M, Fabbi M, Hayashi H, Park N, Attala K, Gu G, French MA, Driver VR (2014). A retrospective cohort study evaluating efficacy in high-risk patients with chronic lower extremity ulcers treated with negative pressure wound therapy. Int Wound J.

[50] Bergersen TK, Storheim E, Gundersen S, Kleven L, Johnson M, Sandvik L, Kvaerner KJ, Ørjasæter NO (2016). Improved clinical efficacy with wound support network between hospital and home care service. Adv Skin Wound Care.

[51] Sundby ØH, Høiseth L, Mathiesen I, Jørgensen JJ, Sundhagen JO, Hisdal J (2016). The effects of intermittent negative pressure on the lower extremities’ peripheral circulation and wound healing in four patients with lower limb ischemia and hard-to-heal leg ulcers: a case report. Physiol Rep.

[52] Sundby ØH, Høiseth LØ, Mathiesen I, Jørgensen JJ, Weedon-Fekjær H, Hisdal J (2016). Application of intermittent negative pressure on the lower extremity and its effect on macro- and microcirculation in the foot of healthy volunteers. Physiol Rep.

[53] Hoel H, Hisdal J (2021). The FlowOx device for the treatment of peripheral artery disease: current status and future prospects. Expert Rev Med Devices.

[54] Holder SM, Dawson EA, Brislane Á, Hisdal J, Green DJ, Thijssen DHJ (2019). Fluctuation in shear rate, with unaltered mean shear rate, improves brachial artery flow-mediated dilation in healthy, young men. J Appl Physiol.

[55] Hoel H, Pettersen EM, Høiseth LØ, Mathiesen I, Seternes A, Hisdal J (2020). A randomized controlled trial of treatment with intermittent negative pressure for intermittent claudication. J Vasc Surg.

[56] Hoel H, Pettersen EM, Høiseth LØ, Mathiesen I, Seternes A, Hisdal J (2021). Lower extremity intermittent negative pressure for intermittent claudication. Follow-up after 24 weeks of treatment. Ann Vasc Surg.

[57] Reyes P, Larée D, Weinstein A, Jara Á (2018). Towards a conceptual model for the use of home healthcare medical devices: the multi-parameter monitor case. PLoS One.

[58] Sekhon M, Cartwright M, Francis JJ (2017). Acceptability of healthcare interventions: an overview of reviews and development of a theoretical framework. BMC Health Serv Res.

[59] B.M.C (2017). ISRCTN registry: Effect of FlowOxTM treatment on healing of lower limb ischaemic ulcers.

[60] Bradshaw C, Atkinson S, Doody O (2017). Employing a qualitative description approach in health care research. Glob Qual Nurs Res.

[61] Braun V, Clarke V (2006). Using thematic analysis in psychology. Qual Res Psychol.

[62] Sandelowski M (2000). Whatever happened to qualitative description?. Res Nurs Health.

[63] Wann-Hansson C, Hallberg IR, Klevsgård R, Andersson E (2005). Patients’ experiences of living with peripheral arterial disease awaiting intervention: a qualitative study. Int J Nurs Stud.

[64] Atkin L, Orsouw M, Bond E (2015). Peripheral arterial disease. Ind Nurs.

[65] Harwood AE, Broadbent E, Totty JP, Smith GE, Chetter IC (2017). “Intermittent claudication a real pain in the calf”—patient experience of diagnosis and treatment with a supervised exercise program. J Vasc Nurs.

[66] Walburn J, Vedhara K, Hankins M, Rixon L, Weinman J (2009). Psychological stress and wound healing in humans: a systematic review and meta-analysis. J Psychosom Res.

[67] Hardiker NR, Grant MJ, Jones I. Self management of long term conditions: a literature review. UK: The Unviersity of Salford; 2013. http://usir.salford.ac.uk/id/eprint/28325/.

[68] Boyce MB, Browne JP, Greenhalgh J (2014). The experiences of professionals with using information from patient-reported outcome measures to improve the quality of healthcare: a systematic review of qualitative research. BMJ Qual Saf.

[69] Aber A, Lumley E, Phillips P, Woods HB, Jones G, Michaels J (2018). Themes that determine quality of life in patients with peripheral arterial disease: a systematic review. Patient.

[70] Janssen AHJ, Mommers EHH, Notter J, De Vries Reilingh TS, Wegdam JA (2016). Negative pressure wound therapy versus standard wound care on quality of life: A systematic review. J Wound Care.

[71] Heikkilä K, Coughlin PA, Pentti J, Kivimäki M, Halonen JI (2019). Physical activity and peripheral artery disease: two prospective cohort studies and a systematic review. Atherosclerosis.

[72] Lambert MA, Belch JJF (2013). Medical management of critical limb ischaemia: where do we stand today?. J Intern Med.

[73] Richards T, Coulter A, Wicks P (2015). Time to deliver patient centred care. BMJ.

[74] Ezeofor VS, Bray N, Bryning L, Hashmi F, Hoel H, Parker D (2021). Economic model to examine the cost-effectiveness of FlowOx home therapy compared to standard care in patients with peripheral artery disease. PLoS One.

