# Supplementary material for: Patient and clinician experiences and opinions of the use of a novel home use medical device in the treatment of peripheral vascular disease - a qualitative study
Source: J Foot Ankle Res. 2021 Dec 3;14:61. doi: 10.1186/s13047-021-00496-2 (PMC8642923; doi:10.1186/s13047-021-00496-2)
Supplement: Supplementary file 1 — Additional file 1. . [file 13047_2021_496_MOESM1_ESM.docx]

**Appendix 1**

**Interview schedule for one to one interviews with patients**

**NB:** the terms “Tell me more about that” and “How did you feel about that?” will be used when interesting topics emerge during the conversation.

**1) Tell me about your experience of using the device**

- - Prompts:
    - Is there anything you found difficult or easy when use the device?
      - Tell me little more about that
        - How did you feel about that?
    - Do you require help with putting the device on and taking it off?
      - Tell me little more about that
        - How did you feel about that?
    - How does using the device fit with your normal daily activities?
      - Tell me little more about that
        - How did you feel about that?

**2) What is your experience of comfort during or after using the device?**

- Prompts:
  - Did you feel any different/odd sensation(s) whilst using the device?
    - - Tell me little more about that
        - How did you feel about that?
  - Did you feel any different/odd sensation(s) after using the device?
    - - Tell me little more about that
        - How did you feel about that?

**3) Do you have any thoughts about how the design might be improved?**

- - - - Tell me little more about that

**4) Did you require any additional treatments for your leg/ foot or pain symptoms during the treatment period?**

- Prompts:
  - Why did you seek these treatments?
    - - Tell me little more about that
        - How did you feel about that?

**5) How has using the device benefitted you?**

- Prompts:
  - Improvement in pain, mobility, appearance of leg/foot, sleep.
    - - Tell me little more about that
        - How did you feel about that?
  - Did you feel any benefits of being directly involved with the management of your condition?
    - - Tell me little more about that
        - How did you feel about that?

**6) If you were to purchase the device what monetary value would you place on it?**

- Prompts:
  - Would your health insurance be helpful for this?
    - - Tell me little more about that

**7) Would you recommend this to other people with the same condition as you?**

- Prompts:
  - If yes or no:
    - - Tell me little more about that
  - If yes: What words would you use to sell this device?
    - - Tell me little more about that

**8) Did any people (family, friends or clinicians) comment on the device?**

- - - - Tell me little more about that

**Question schedule for focus group with clinicians**

**1)** **What are your general opinions about the device and its clinical effects?**

- - - - Tell me little more about that
        - How did you feel about that?

**2) What do you think the Minimal Clinical Important Difference (MICD) should be regarding wound healing with this type of treatment?**

- Prompt:
  - What clinical endpoints do you see as being important?
    - - Tell me little more about that
        - How did you feel about that?

**3) What do you think the MICD should be regarding improvements in haemodynamic outcome with this type of treatment?**

- Prompt:
  - What clinical endpoints do you see as being important?
    - - Tell me little more about that
        - How did you feel about that?

**4) What patients groups do you think could benefit from this treatment?**

- - - - Tell me little more about that
        - How did you feel about that?

**5) What are your opinions on the implementation of novel treatments (such as this one) in your clinics (including key decision makers in the Norway health service)?**

- - - - Tell me little more about that
        - How did you feel about that?

**6) What are your opinions on reimbursements regarding the use of FlowOx™ in the health service in Norway?**

- - - - Tell me little more about that
        - How did you feel about that?

**7) What do they see as a critical price point for the device in relation to the anticipated (or observed) clinical benefit?**

- - - - Tell me little more about that
        - How did you feel about that?
